# Bilateral pulmonary emboli in an amateur kick boxer: A case report and literature review

**DOI:** 10.1002/ccr3.4733

**Published:** 2021-08-30

**Authors:** Elabbass A. Abdelmahmuod, Aseel Alzibdeh, Ali Rahil

**Affiliations:** ^1^ Department of Internal Medicine Hamad Medical Corporation Doha Qatar

**Keywords:** pulmonary embolism, pulmonary embolism, deep vein thrombosis, trauma, kick boxer

## Abstract

Increased knowledge of unusual pulmonary embolism presentations in athletes will prevent delays in the diagnosis and management.

## Graphical abstract

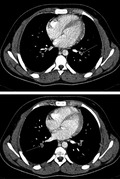

## 1 INTRODUCTION

Pulmonary embolism in teenagers is rare and often goes undiagnosed, so a high suspicion is warranted. To avoid mortality and morbidity, pulmonary embolism remains a condition that requires strong clinical suspicion. Moreover, the worry is very low in young, stable individuals relative to older individuals with multiple co‐morbid conditions. This paper reports a 16‐year‐old previously healthy boy admitted to the medical unit with chest pain following blunt trauma to the chest resulting from a kickboxing training session diagnosed with bilateral pulmonary infarcts due to pulmonary embolism. A workup for the secondary causes was unrevealing. He was managed with subcutaneous enoxaparin and then switched to warfarin. His clinical course over the 4‐month period of follow‐up was uneventful. Increasing awareness among healthcare providers, including sports medicine professionals, of atypical presentations of venous thromboembolisms in student‐athletes may decrease the underestimation of diagnosis among this population in the hope of prompting the required additional testing to prevent a delay in diagnosis.

Pulmonary embolism affects over one in one thousand Americans every year. It has a mortality of more than 15% in the first 3 months after the diagnosis.^1^

Young athletes without the usual risk factors or genetic predispositions are considered a low‐risk population to develop venous thromboembolism (VTE). However, there seems to be an increasing number of reports describing deep vein thrombosis (DVT) or pulmonary embolism (PE) in otherwise healthy endurance athletes.^2^

There is no clear association between trauma and pulmonary embolism, but the theory behind the relationship that heavy and intense exercise is associated with severe dehydration and hemoconcentration that aggravate platelet adhesions on top of vessel injury secondary to trauma and lead to thrombosis and clot formation.^3^

The most common risk factors include older age, immobility, inherited clotting disorders, post‐operative states, and smoking history. However, PE secondary to direct blunt trauma to the chest was not reported.

Here, we present a 16‐year‐old male athletic adolescent who developed a large bilateral pulmonary embolism after blunt trauma to the chest during a kickboxing class.

## 2 CASE REPORT

A 16‐year‐old male athlete presented to the emergency department with a 2‐h history of pleuritic left chest pain after blunt trauma to the chest during a kickboxing training session 4 days prior to the presentation. On arrival at the hospital, he was tachycardic at 115/min, blood pressure was 119/68 mmHg, respiratory rate was 21/min, oxygen saturation was 98% on room air and temperature was 38°C, the rest of physical examination was unremarkable.

**FIGURE 1:**
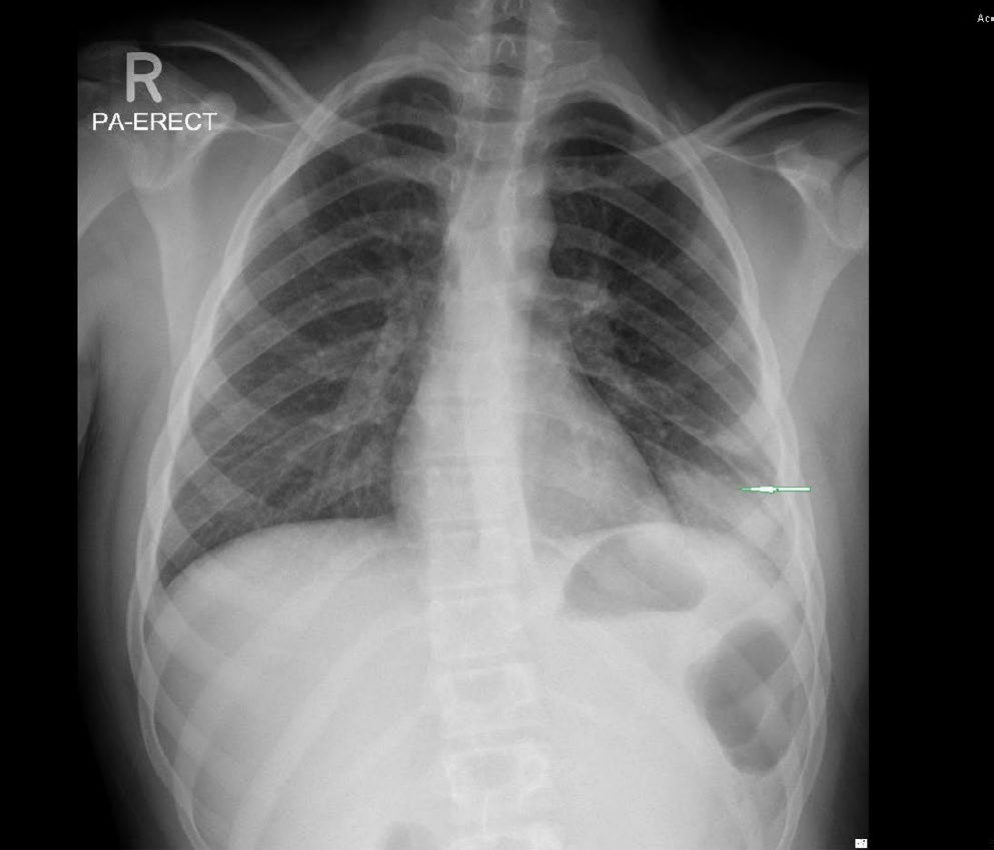
CXR showed opacity in the left lower lung zone with evidence of small effusion

Chest radiography (sees Figure 1) showed opacity in the left lower lung zone with evidence of small effusion, however, no evidence of rib fracture.

**FIGURE 2:**
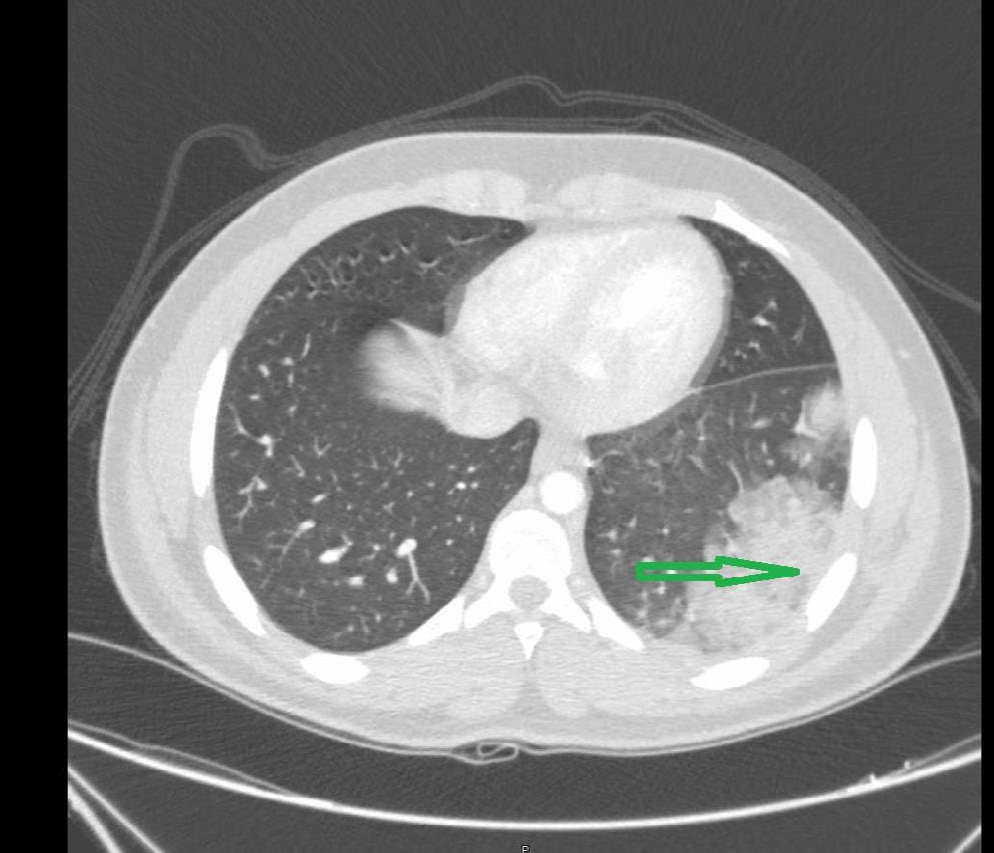
CT chest showed ‘left lower lobe opacities that may be secondary to lung contusion/alveolar hemorrhage’

CT chest (See Figure 2) showed ‘left lower lobe opacities that was thought to be related to lung contusion/alveolar hemorrhage’. He was admitted with community‐acquired pneumonia for 2 days, treated with antibiotics, and was discharged home.

At home, his condition worsened, and he had started to experience severe chest pain. He denied pain or swelling of his lower limbs, he denied extended periods of immobilization, recent surgery, use of anabolic steroids, current or past history of malignancy, constitutional symptoms or family history of thrombophilia or recurrent pregnancy loss. Three days after discharge, he presented to a primary hospital with hemoptysis. Physical exam was significant for tachypnea at 30 respirations/min.

**FIGURE 3:**
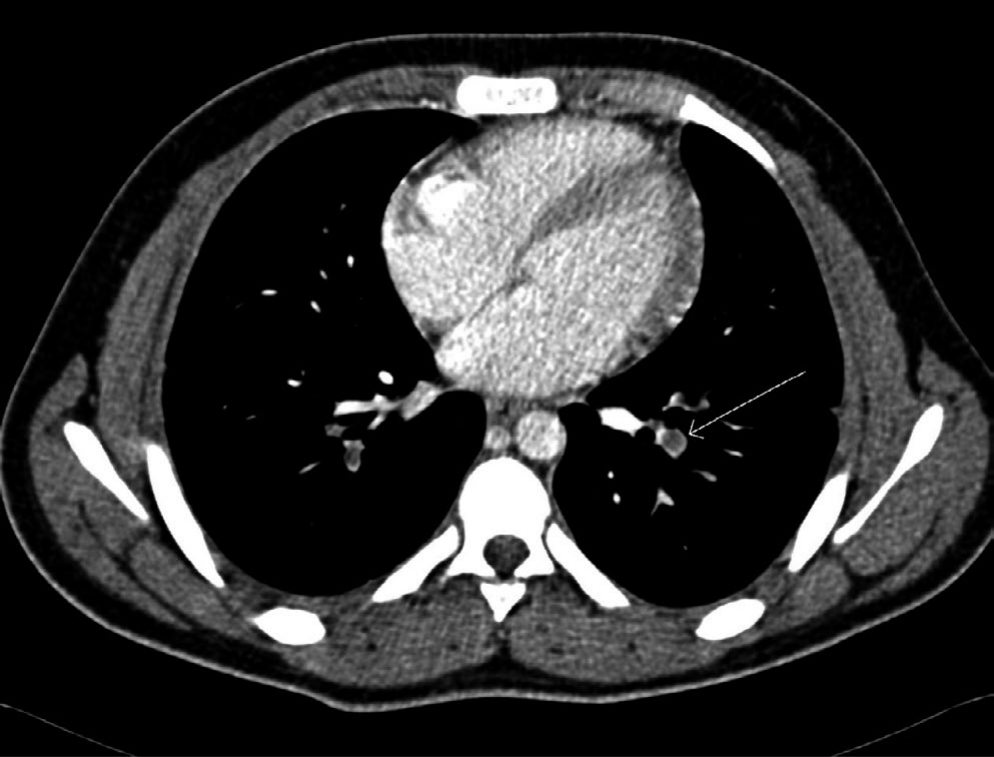
CT chest showed left side pulmonary embolism

**FIGURE 4:**
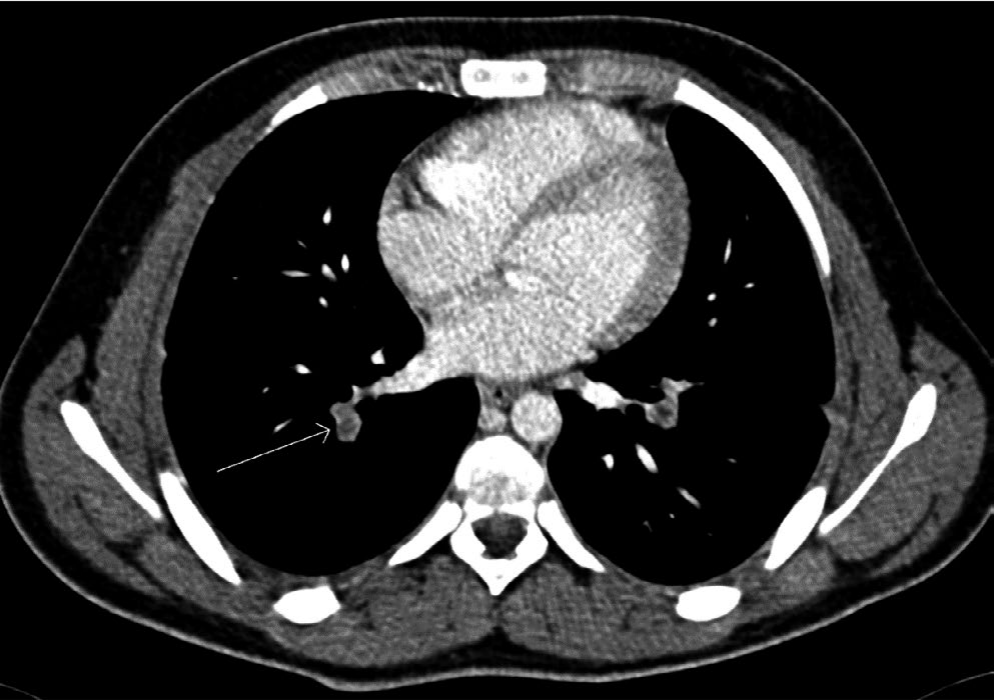
CT chest showed right side pulmonary embolism

Repeated CT chest showed Bilateral PE with pulmonary infarction (See Figure 3). The CT scans were reviewed with a senior radiologist and he noted the presence of an early PE on the initial CT scan (see Figures 3 and 4). He also noted a large lymph node (8 mm). Doppler ultrasonography of lower limb showed patent venous system with no evidence of deep vein thrombosis.

Because of the enlarged Lymph node on the CT thorax, we investigated for underlying lymphoma as the underlying cause of PE, despite the absence of the usual clinical features. However, a CT of his abdomen did not reveal any evidence of lymphadenopathy or organomegaly. Thrombophilia workup was negative (see Table 1).

**TABLE 1:** Reveled negative thrombophilia workup

Detail	Value w/Units	Flags	Normal range
Thrombin time	16.3 s	Low	16.5–20.7
Lupus confirm	42.1 s	Normal	27.7–43.5
Protein C activity	72.0%		70.0–140.0
Protein S activity	107.6%		72.0–126.0
ATAC	100.2%		79.4–112.0

He was treated with subcutaneous enoxaparin then switched to warfarin with uneventful hospital course.

He completed 3 months of anticoagulation after which a thrombophilia workup was negative.

## 3 DISCUSSION

Pulmonary embolism (PE) is a disorder characterized by thrombus occluding the pulmonary artery that usually occurs secondary to thrombus originating from deep veins (DVT).^4^ The underlying.

The Virchow triad explained the pathogenesis of PE, which includes hypercoagulability, hemodynamic changes (stasis, turbulence), and endothelial injury/dysfunction.^5^

Our case is interesting from two aspects: The first is that it occurred in adolescents, an unusual age group for PE. On the contrary, is that it occurred following minor blunt trauma to the chest.PE in adolescents is rarely seen in clinical practice.^6^ However, the disease is not as rare when postmortem examination is reviewed.

The incidence of PE in a retrospective study involving children and adolescents aged 0–19 years was 3.7% based on the autopsy data with a mean age of 8 years, and trauma represented 8.4% of predisposing factors.^7^ Venous thromboembolism in children in a Hong Kong study by Lee et al. was found to be at a crude annual rate of 0.74 per 100,000 children.^8^ Predisposing factors were hereditary thrombophilia conditions like protein C and protein S, anti‐cardiolipin antibodies, recent surgery, malignancy, and sepsis. However, central venous catheterization was the most common. Ho et al. reported a case of DVT and PE in a 14 years old in Hong Kong who was previously healthy following minor trauma and osteomyelitis.^9^ In a study by Menaker J et al. that included 94 patients with pulmonary embolism secondary to trauma, chest trauma represented 37% of cases, and 15% of emboli were diagnosed within 2 days and 37% within 4 days.^10^

The diagnosis can be easily missed and delayed in this age group. The diagnosis was entertained in 15% of patients proved later to have PE.^11^ Wells score, Geneva score, and the pulmonary embolism rule‐out criteria (PERC) have not been validated in children. However, D‐dimer testing has the diagnostic ability in children.^12^

Well's score had a 100% failure rate for triaging athletes with known DVT/PE, as reported by Zaleski A. The score had limited utility for recognition of VTE in athletes, contributing to missed or delayed diagnosis.^13^ This was the case for our athlete.

There were several published case reports of previously healthy athletes who developed DVT/PEs. However, in contrast to our patient, these reports included athletes who had one or more risk factors for VTEs such as OCP use, anabolic steroid abuse, or later were found to have a protein C deficiency.^14^

The usual association between trauma and DVT or PE is decreasing the mobility of the patient and lead to venous stasis. However, the direct relationship between kickboxer trauma and PE is not well understood and could be secondary to vessel injury that predisposes to thrombus formation and PE.^15^

Upon literature review, there is only one case reported the association between trauma and PE in 2013 a young female presented 1 h after a motor vehicle accident with chest pain and turned to be PE. However, she was using contraceptive pills.^16^

## 4 CONCLUSION

Increasing awareness among healthcare providers, including sports medicine professionals, of atypical presentations of venous thromboembolisms in student‐athletes, may decrease the underestimation of diagnosis among this population in the hope of prompting the required additional testing to prevent a delay in diagnosis.

## CONFLICT OF INTEREST

None declared.

## AUTHOR CONTRIBUTION

EA involved in writing and editing. AA involved in writing and editing. AR involved in writing, editing, and final approval.

## ETHICAL APPROVAL

This case was approved by the Hamad Medical Corporation's Medical Research Center, and the patient consented to the publication of his case.

## Data Availability

Data and materials are available on reasonable request.
